# What Volcanoes Are Not

**Published:** 1881-11

**Authors:** 


### WHAT VOLCANOES ARE NOT.

"What is a volcano?" This is a familiar question, often addressed to us in our youth, which "Catechisms of Universal Knowledge" and similar school manuals have taught us to reply to in some such terms as the following: "A volcano is a burning mountain, from the summit of which issues smoke and flames." This description, says Professor Judd, is not merely incomplete and inadequate as a whole, but each individual proposition of which it is made up is grossly inadequate and, what is worse, perversely misleading. In the first place, the action which takes plaee at volcanoes is not "burning," or combustion, and bears, indeed, no relation whatever to that well-known process. Nor are volcanoes necessarily "mountains" at all; essentially, they are just the reverse—namely, holes in the earth's crust, or outer portion, by means of which a communication is kept up between the surface and the interior of our globe. When mountains do exist at centers of volcanic activity, they are simply the heaps of materials thrown out of these holes, and must, therefore, be regarded not as the causes but as the consequences of volcanic action. Neither does this action always take place at the "summits" of volcanic mountains when such exist, for eruptions occur quite as frequently on their sides or at their base. That, too which popular fancy regards as "smoke" is really condensing steam or watery vapor, and the supposed raging "flames" are nothing more than the glowing light of a mass of molten material reflected from these vapor-clouds. The name of volcano has been borrowed from the mountain Vulcano, in the Lipari Islands, where the ancients believed that Hephæstus, or Vulcan, had his forge. Volcanic phenomena have been at all times regarded with a superstitious awe, which has resulted in the generation of such myths as the one just mentioned, or of that in which Etna was said to have been formed by the mountains under which an angry god had buried the rebellious Typhon. These stories changed their form, but not their essence, under a Christian dispensation, the Vulcano became regarded as the place of punishment of the Arian Emperor Theodosius, and Etna as that of Anne Boleyn, who had sinned by perverting the faith of King Henry VIII.—*From " Volcanoes, their Action and Distribution," in Popular Science Monthly for November.*

